# Medical students’ perceptions towards research at a Sudanese University

**DOI:** 10.1186/s12909-016-0776-0

**Published:** 2016-09-29

**Authors:** Tarig Osman

**Affiliations:** Graduate College, University of Medical Sciences and Technology, Khartoum, Sudan

**Keywords:** Sudan, Medical students, Attitudes, Research

## Abstract

**Background:**

Inadequately designed curricula and barriers to research have not enabled students to realize the crucial importance of research to clinical practice. Several studies have reported variable results with regards to research training. The aim of this survey was to evaluate research training at a university in Sudan which had provided research training for 17 years.

**Methods:**

A university-based survey design, using a self-administered questionnaire, was conducted among final year medical students who completed 2 years of research training. Descriptive frequency analysis and bivariate analysis were performed using IBM SPSS version 20.

**Results:**

The response rate was 76 %. Ninety-five (91.3 %) perceived research important to medicine and 62 (59.6 %) perceived that it should be a requirement for partial fulfillment of the MBBS degree. Elevation of professional standing as a clinician was the prime reason for pursuing a career in research (18/68.2 %). Main reasons for not pursuing a career in research was research being time consuming (49/59.8 %) and focusing on clinical service being more important than clinical research (48/58.5 %). Fifty-five (56.1 %) believe that their supervisor gave them a positive attitude to research. Principle barriers to student research were lack of funding (75/72.1 %), insufficient time (71/68.3 %) and the demands of the curriculum (70/67.3 %). No significant differences were detected between gender and perceptions towards research.

**Conclusion:**

The curriculum should be revised to address the gaps in research training. A small group learning model should be adopted to train students in research and provide supervision to group research projects. This model would improve academic learning, skills acquisition, encourage student interest in research, reduce barriers to student research and make better use of limited resources.

## Background

Research training is a critical component of medical school education [1, 2]. Medical schools are expected to train students in research to meet accreditation standards, to support students’ career prospects and to generate a pool of researchers. It is important that tomorrow’s clinicians are equipped with adequate research training during their undergraduate studies to promote critical thinking, develop critical appraisal skills and become research-oriented. Research experience is strongly linked to postgraduate research initiatives and future career achievements [3–6]. An American study revealed that 97 % of students considered research a useful alternative to electives [7].

Medical schools have reformed curricula to train students in research. Gaps in research training are due to inadequate approaches, shortage of research staff, lack of funds and scarce infrastructure for research training. Anecdotal reports from theses examiners in Sudan indicate that the majority of students had inadequate knowledge of research indicating insufficient exposure to research training.

Most students are not aware of why research is crucial to health care [3]. Negative attitudes toward research serve as an obstacle to learning associated with poor performance in research [8]. Attitude to, knowledge of and barriers toward research are three key factors that have an impact on research success [9]. The perception of undergraduate students toward research is unknown in Sudan. The objective of this study was to explore students’ perceptions; attitudes; motives and barriers toward research; and their perceptions on the quality of research supervision. The findings of will assist in identifying gaps in the research training and suggest means for improving research training.

## Methods

Proper medical research in Sudan dates back to 1902, when Mr. (later Sir) Henry S. Wellcome (the founder of the Wellcome Foundation) donated equipment of a research laboratory together with a library and museum as a generous gift to the Government of Sudan [10]. The Welcome Tropical Research Laboratories were established as part of Gordon Memorial College and conducted pioneer research which contributed to the improvement of environmental health, control of endemic diseases such as leishmaniasis, schistosomiasis and malaria, and to the improvement of health services [11]. Throughout the past decades the responsibility of medical and health research has passed from government institution to another. Currently health research in Sudan is a duty of the Research Directorate at the Federal Ministry of Health, and the faculties of medicine and health sciences at the universities. There are more than thirty specialized institutions, governmental and non-governmental, involved in health research. Health research priorities cover all the domains of health research, in particular biomedical, clinical, epidemiology and health system research as well [12]. Given the rich history of research in Sudan, an emerging threat is the emigration of qualified research experts from various domains in medical and health research, due to the dire economic consequences. This would adversely affect the quality of research training in university and research institutes.

This cross-sectional study was conducted at the University of Medical Sciences and Technology in July 2015. The University was established in 1996 and students graduate after 5 years of intensive teaching and training in medicine. Research training was formally introduced to the first medical batch in their fourth academic year in 1999 and was provided continuously to consecutive batches. Research methodology and biostatistics lectures are taught over one academic year. In the following year, students undertake a research project under supervision and afterwards submit a thesis. Supervisors not affiliated to the university may supervise student research Students may conduct clinical research, public health research and epidemiological research. To date there are 18 batches that have completed research training. The research methodology curriculum has undergone several changes. There is no published study to date that assessed the context of research training provided to medical students.

The study population was medical students in their final fifth academic year. All 136 final year students were eligible for inclusion into the study to provide meaningful analysis. This population completed 2 years of research training during which it attended sessions on research methodology and biostatistics; have prepared individual proposals; and conducted research individually under supervision. Each student would submit an individual thesis. No two students are allowed to work on the same topic and submit the same thesis. The study population includes a combination of students from Sudan and Sudanese students from Gulf States, Europe and North America. The majority of the medical graduates migrate to Gulf States, Europe and North America either after graduation or after a short period of internship. The primary research instrument was a self-administered coded questionnaire constructed from variables obtained from research literature on the topic. The purpose of the questionnaire was to assess five conceptual concepts identified in the literature: perceptions to research (5 questions); motives for conducting research in school (3 questions); reasons for pursuing a career in research (5 questions); reasons for not pursuing a career in research (9 questions); and barriers to student research at the university (13 questions). Competency of research supervisor (10 questions) was an additional fifth theoretical concept added to the questionnaire.

The questionnaire was not pre-tested for fear of contamination and testing threat as there is no comparable batch and the study population was relatively small. Data was analyzed using IBM SPSS version 20. Descriptive frequency analysis was performed for all variables in the questionnaire. Chi-squared tests were conducted to investigate the association between gender and perceptions to clinical research. The statistical level of significance was a *p* value less than 0.05. Cronbach alpha for the reliability of the questions on perception towards research and the research supervisor were 0.70 and 0.80 respectively.

## Results

The response rate was 76 % and the reasons were absence of the students at the time of the survey, refusal to participate and exclusion of incomplete and inconsistent questionnaires from analysis. Of the 104 students who completed the survey, 45 (43.3 %) were males and 59 (56.7 %) were females. The mean age ± SD was 22 ± 1.4 years.

The majority of students perceived research important to the practice of medicine (95/91.3 %), should be part of the medical curricula (79/76 %) and conducting research during medical school is important (78/75 %). Less students perceived having research experience an important criterion for residency training in medicine after graduation (66/63.5 %) and submission of a thesis be a requirement for partial fulfillment (62/59.6 %) of the MBBS degree.

The principle motives to conduct research in medical school were: it is mandatory in the curriculum (84/80.8 %), to facilitate acceptance to a residency program after graduation (66/62.5 %) and to pursue a career in research (22/21.2 %). The main reasons for pursuing a research career were that it will elevate professional standing as a clinician and there are no fixed hours (Table 1). The main reasons for not pursuing for pursuing a research career were research is time consuming, focusing on practicing clinical service is more important than research and research is stressful.

**Table 1:** Reasons for pursuing a career in research and not pursuing a career in research

	F	Percent
Reasons for pursuing a career in research (*n* = 22)		
I want a career in research because it will elevate my professional standing as a clinician.	18	68.2
I want a career in research because there are no fixed working hours.	14	63.6
I want a career in research because I enjoy the pleasure of doing research.	12	54.5
I want a career in research because there are monetary and financial benefits.	10	45.5
I want a career in research because there are no emergency, clinical duties and on-calls.	8	36.4
Reasons for not pursuing a career in research (*n* = 82)		
I don’t want to pursue a career in research because research is time consuming.	49	59.8
I don’t want to pursue a career in research because I believe focusing and practicing my clinical profession is more important than research.	48	58.5
I don’t want to pursue a career in research because research is stressful.	47	57.3
I don’t want to pursue a career in research because I didn’t receive a positive attitude towards research during university.	40	48.8
I don’t want to pursue a career in research because I don’t like research.	37	45.1
I don’t want to pursue a career in research because research is difficult and complex.	32	39
I don’t want a career in research because there are no monetary and financial benefits.	21	25.6
I don’t want a career in research because it will not elevate my professional standing as a clinician.	14	17.1
Other reasons.	9	11

Ninety-eight (94.2 %) students had acknowledged conducting research with a supervisor. Students’ responses with regards to the competency and commitment of the research supervisor were: supervisors possess both knowledge experience and research skills to assist in research (67/64.4 %); supervisors were available and easy to reach (55/56.1 %); supervisors provided constructive feedback to improve their proposal and thesis (55/56.1 %); would work with the same supervisor again (49/50 %); would have been able to do research without the guidance of a supervisor (40/40.8 %); supervisors gave adequate supervision to independently and confidently write a successful research proposal after graduation(46/46.9 %); can confidently produce a thesis after graduation (42/42.9 %) and were stimulated to publish (35/35.7 %). Overall, 55 (56.1 %) believe that their supervisors gave them a positive attitude towards research.

The most commonly reported barriers to research were lack of adequate funding; insufficient time; and the medical curriculum is very demanding (Table 2). No significant associations were found between sex and perceptions towards clinical research (Table 3).

**Table 2:** Barriers to student research at the university (*n* = 104)

	F	Percent
Lack of adequate funding for student research.	75	72.1
Time allocated to student research is insufficient.	71	68.3
The medical curriculum is very demanding.	70	67.3
Difficulty in following up patients or research subjects.	67	64.4
Lack of supportive staff such as biostatisticians, bio-ethicists and proof editors.	63	60.6
Lack of interest in research by faculty.	59	56.7
Lack of well-equipped laboratory facilities.	61	58.7
Lack of adequate research and biostatistics curriculum.	55	52.9
Lack of competent and committed supervisors.	53	51
Lack of well-equipped computer facilities.	47	45.2
Lack of study subjects or samples for research.	38	36.5
Difficulty in obtaining administrative approval.	37	35.6
Difficulty in obtaining ethical approval.	32	30.8

**Table 3:** Relation between gender and perceptions towards research (*n* = 104)

	Males (Yes)	Females (Yes)	*P* value
Is research important to the practice of medicine?	42 (93.3 %)	53 (89.8 %)	0.529
Is conducting research by students during medical school important?	32 (71.1 %)	46 (78 %)	0.424
Should research training be part of the medical curricula?	34 (76.3 %)	45 (76.3 %)	0.933
Should submission of a thesis be a requirement for partial fulfillment of the MBBS degrees?	25 (55.6 %)	37 (62.7 %)	0.461
Should having research experience be an important criterion for residency training in medicine after graduation?	25 (55.6 %)	41 (69.5 %)	0.144

## Discussion

### Perceptions towards research

The results reveal similarities and contrasts with a Saudi study which found medical students had higher favorable perceptions to the importance of research methodology to the curriculum (91.9 %), medical field (97.1 %) and conducting research during school (87.1 %) [3]. In contrast, a greater proportion of the students compared to the Saudi study (43 %) perceived research experiences to be an important part of acceptance to residency. This could be explained by the fact that most students would emigrate to pursue specialist training in the United Kingdom, Ireland and North America. Competition for residency posts in these countries is also based on having adequate research knowledge and training. Attitudes and the level of interest in research are influenced by subject curiosity and factors such as barriers to research [9]. The students had varied perceptions. Perceptions with regards to submission of a thesis being a requirement for partial fulfillment of the MBBS degree and residency training did not compare with perceptions of the importance of research to the practice of medicine, conducting research during medical school and research training. This would suggest that there is a need for introductory sessions to teach students the importance of research to medicine. The sessions should enable students to understand why research is incorporated in the medical curriculum; why they are undertaking research and submitting a thesis; and how research experience would be important for residency training after graduation.

### Motives

The prime motive to conduct research because it is mandatory in the curriculum is comparable to the Saudi study (78.5 %) [3]. Greater proportions of Saudi (82.9 %) and Canadian students (43 %) revealed their key motive for doing research was to facilitate acceptance to a residency program [3, 8]. Expectedly a low proportion of students in this survey wanted to pursue a career in research. The nature of research is challenging and does not appeal to many people, despite its gratifying rewards when successfully completed. To encourage more students to pursue a career in research requires that the University review the methodology of research education and training. Barriers to research training are mostly amenable at the University level. Unfortunately, in Sudan the common and negative general notion among parents and the community is that medical students are expected to become high earning clinicians to live an affluent life; which further deters students from pursuing a career in research. One suggestion to motivating students to pursue a career in research would be to build relationships between research centers and the university. Supervised visits will allow students to observe a genuine research environment and to appreciate the role of research in human health. Researchers from such centers can act as supervisors and provide supervision to students to investigate more relevant topics. This would further generate students’ interest in research and maybe pursue a career in research.

### Role of research supervisors

Competent research supervisors are critical in motivating students to become research-oriented clinicians. The Saudi study reported 84.7 % of students cited the lack of professional supervisor as a barrier to research [3]. University supervisors are usually over-burdened with academic and clinical work and many lack sufficient research experience to provide adequate research supervision. The number of scientists over the past two decades has declined internationally and clinical researchers are critically needed [1, 8, 13]. One recommendation would be to shift from individual student supervision to small group supervision. “More hands make for lighter work” and “two heads are better than one.” Both students and supervisors can accrue benefits from group supervision [14]. This technique would allocate a small number of students to the few but more capable supervisors. Group experiences contribute positively to student learning, skills acquisition, retention and overall college success which is important in the professional world [15–18]. The small group technique can be expanded to become a learning model. It can provide academic tutoring in the form of tutorials or seminars; as well as group research project supervision. The technique can build student confidence and build motivation. It would enable students to transform research knowledge into research practice. Research is best conducted in a group and students can learn from sharing ideas and discussing different perspectives. There is no guarantee that all benefits will be gained from small group learning model. Often group projects backfire badly when not designed, supervised, and assessed in a manner that promotes meaningful teamwork and collaboration [14]. A proper group model may increase student numbers to pursue a career in research, alleviate disadvantages associated with lecturing large student batches and is more practical when there is a large student-teacher ratio. If a small group model is conducted properly, students would develop a genuine interest in research and not see research as an academic burden that they are obliged to fulfil. Supervisors provide valuable learning and training through group supervision. Supervisors can create a positive attitude and provide guidance for students to be more aware of health problems within their society [19, 20].

### Barriers to research

The barriers reported are similar to those reported in the literature [1, 3, 8, 9, 13, 19, 21–34]. Surprisingly, 52.9 % of students cited the lack of adequate research and biostatistics curriculum as a barrier to research, suggesting that the curriculum requires critical examination. Essential prerequisites for any study are adequate knowledge of the study subject and awareness of research principles [9]. The main challenges facing clinical research are lack of trained manpower and inadequate funding [34]. The University provides research funds but the amounts do not suffice and most students are left to spend from personal finances. Institutions should seek funds for student research [9]. This may not be feasible as funds may take considerable time to be secured and depend on good quality proposals. A common barrier reported by the students is the lack of time allocated to research. The university medical curriculum is physically and mentally demanding and the frequent clinical exams force students to prioritize the major demands of the curriculum ahead of research activities. These circumstances will result in a decreased interest to conduct research. Allocating a fixed-time in the academic calendar for student research may minimize the time obstacle and enable more interaction between students and their supervisors. Recruiting research support staff can provide additional awareness of practical application of research. Motivating students’ research activity can fill the void of clinician scientists and help developing countries to achieve self-reliance in health research [35]. The small group learning model may also reduce the barriers to student research (e.g. funding, obtaining ethical and administrational approval, finding study subjects) and would make better use of available but limited resources (e.g. human, time and infrastructure).

The study findings are limited to the university. Self-reporting may result in respondent bias and the response rate may result in possible selection bias. The yes/no construct of the questions may have limited true variance in responses. The external validity also applies to the period of research training during which this batch was exposed and the results do not reflect past students’ perceptions towards research.

## Conclusion

Students had variable perceptions towards research. The findings of this survey can provide insights to improving research training. Gaps should be addressed to improve perceptions, stimulate interest in pursuing research careers and to uphold the university’s standing as a provider of credible research training. A small group learning model should be assessed if it would improve perceptions, limit barriers, motivate students and improve the quality of supervision. The curriculum should be revised and further studies would need: (1) to examine student theses to identify inadequacies in knowledge, skills and supervision; (2) to investigate supervisors’ knowledge and research capacity to provide adequate supervision; and (3) to investigate the impact of supervisors’ academic and clinical workload on student supervision.
